# Recurrent bicycle falls by an elderly man who developed multiple intramuscular abscesses associated with urine leakage secondary to posterior urethral injury caused by pelvic ring disruption

**DOI:** 10.1002/ccr3.7576

**Published:** 2023-06-19

**Authors:** Yoko Matsumoto, Keisuke Tanno, Yuhei Nakamura, Kohei Hamamoto, Hanako Yoshihara, Takahiko Fukuchi, Noriko Oyama‐Manabe, Hitoshi Sugawara

**Affiliations:** ^1^ Division of General Medicine Department of Comprehensive Medicine 1 Jichi Medical University, Saitama Medical Center Saitama Japan; ^2^ Department of Radiology Jichi Medical University, Saitama Medical Center Saitama Japan

**Keywords:** bicycle falls, geriatric, geriatric cyclists, pelvic dislocation, pelvic fracture, urethral injury

## Abstract

**Key Clinical Message:**

With the aging of the population, physicians need to pay more attention to assessing the presence or absence of pelvic fractures and urinary retention associated with urethral injury due to such fractures in the elderly when falling from bicycles.

**Abstract:**

Walking ability does not rule out the presence of pelvic fractures. Many geriatric patients are likely to fall off bicycles. Physicians should pay more attention when assessing complications related to urethral trauma caused by pelvic fractures in the elderly after falling from bicycles.

## 1 CLINICAL CASE

**FIGURE 1:**
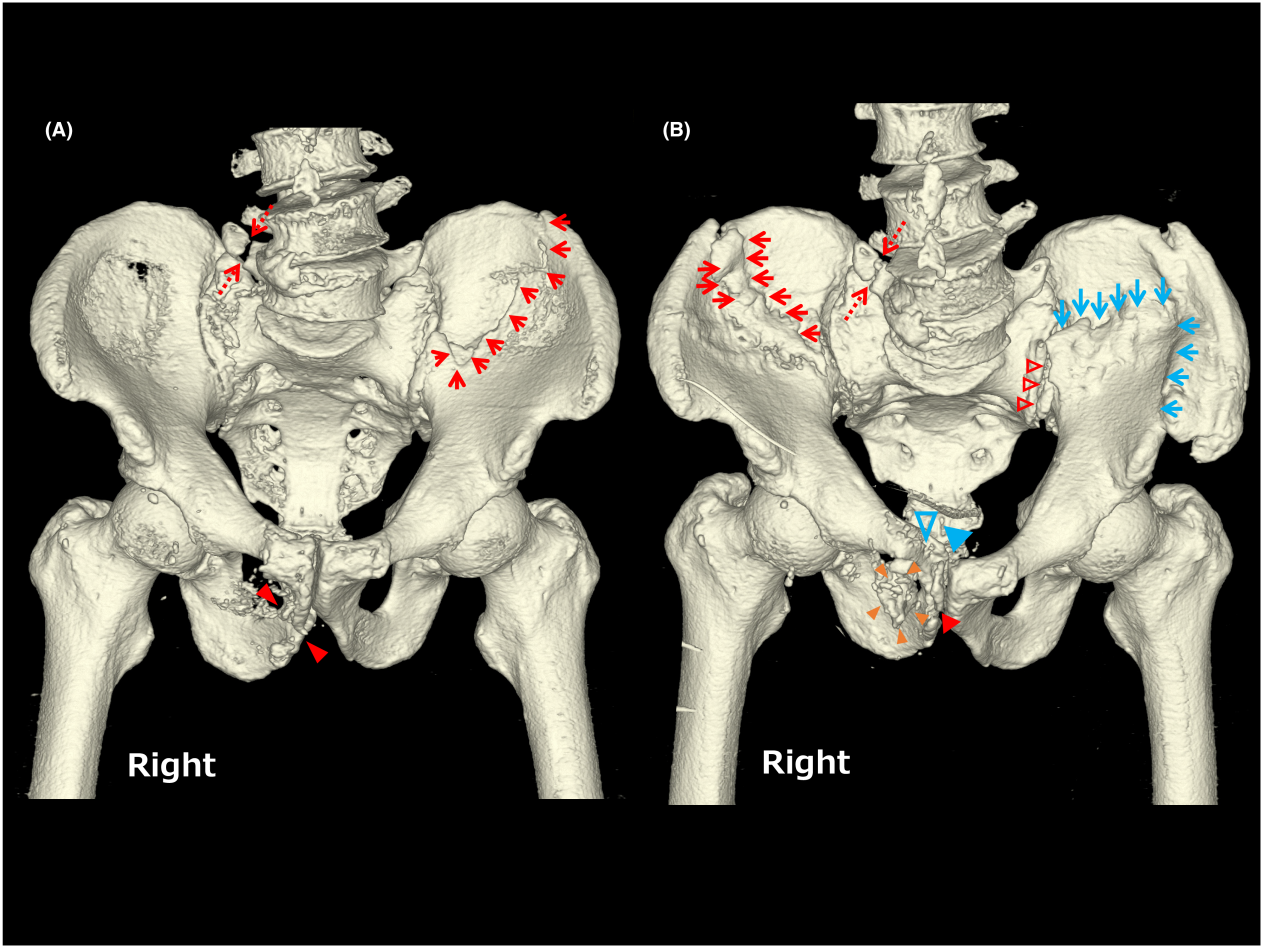
A and B show the frontal 3D computed tomography reconstruction of the abdominopelvic computed tomography (CT). A was taken 6 months before admission and incidentally revealed pelvic fractures of the left ilium (red arrows), right costal process of the fifth lumbar vertebra (red dotted arrows), and right ischium (red arrowheads), but no dislocation of the pelvic ring. In contrast, B was taken on admission when CT‐guided catheter‐inserted drainage of the intermuscular abscesses was performed. It revealed fragility pelvic fractures with new fracture sites and pelvic ring disruption: right ilium (red arrows), deviated left ilium (blue arrows), right costal process of the fifth lumbar vertebra (red dotted arrows), left sacrum (red open arrowheads), deviated right ischium (red arrowhead), detached right ischium (dark yellow arrowheads), right pubis (blue open arrowhead), and pubic symphysis diastasis (blue arrowhead).

**FIGURE 2:**
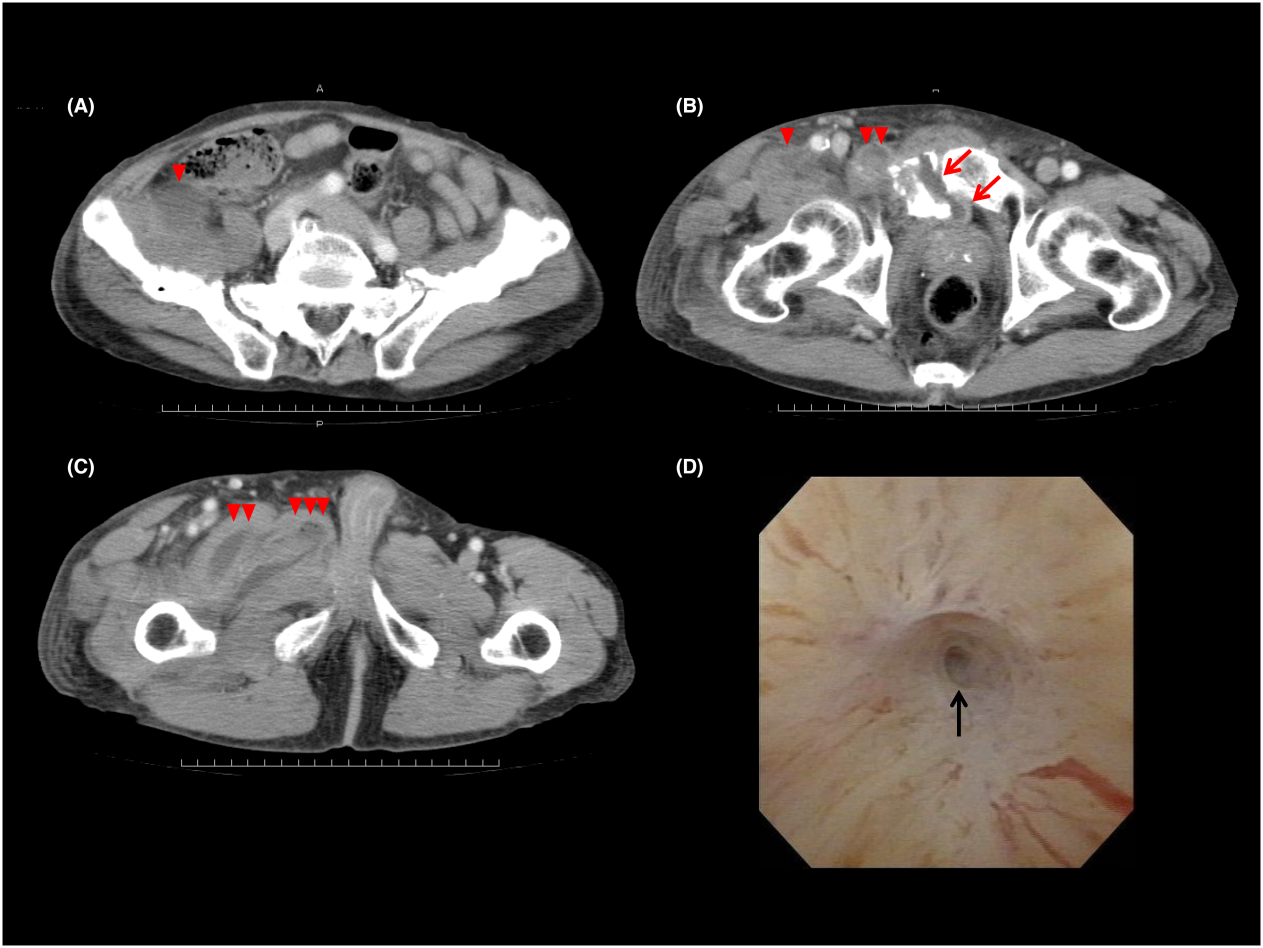
A–C shows the contrast‐enhanced pelvic computerized axial tomography performed 3 days before admission. These images reveal multiple right intramuscular abscesses, including the iliacus muscle (A–C; single red arrowhead), pectineus muscle (B and C; double red arrowheads), abductor brevis muscle (C; triple red arrowheads), and abscess of the pubic symphysis (C; red arrow). D shows the cystoscopy performed 11 months after transfer. Complete obstruction (black arrow) of the distal side of the membranous urethra.

A 79‐year‐old man presented to the emergency department with redness, swelling, and pain in his right hip and thigh, lower abdominal distention due to urinary retention, and fever. He had a history of falls while riding his bicycle over the past 4 years. Nine months before admission, he visited his primary care clinic for left buttock and thigh pain. The patient had never previously reported a bicycle fall. Pelvic radiographs taken at that time showed no signs of fracture. Six months prior, he had been hospitalized for melena caused by acute colonic diverticulitis, during which an abdominopelvic computed tomography (CT) (Figure 1A) incidentally revealed pelvic fractures (Rommens IIc^1^), but no dislocation of the pelvic ring. The patient was able to walk and was discharged after two days. Five months prior, he had fallen off his bicycle and was still working a light labor job. Two weeks prior, he had another bicycle fall but managed to walk afterward. However, the patient noted residual urine. Six days prior, he started experiencing increasing pain in his right hip and thigh. Three days later, a contrast‐enhanced pelvic CT performed by the previous clinic revealed fragile pelvic fractures (Rommens IVc^1^) with new fracture sites (Figure 1B), resulting in pelvic ring disruption and multiple intramuscular abscesses in the right hip region (Figure 2A–C).

On admission, the patient was in distress, and physical examination showed tenderness of the left lower distended abdomen, point tenderness at the pubic symphysis, redness, swelling, warmth from the right hip joint through the right lower leg to the right ankle joint, and pitting edema. An attempt to insert a Foley catheter on hospital day (HD) 1 failed because cystoscopy could not identify the internal urethral orifice due to a tear in the prostatic urethra. As urethral reconstruction had to be abandoned, the patient underwent percutaneous cystostomy. CT‐guided catheter‐inserted drainage of the intermuscular abscesses drained fluid that smelled like urine, and in which **e**xtended‐spectrum beta‐lactamase‐producing *Escherichia coli* was detected by culture on HD 7. He was treated with 2 g cefmetazole every 8 h for 4 weeks and was then transferred for rehabilitation while wearing a pelvic belt on HD 48. Cystoscopy performed 11 months after the transfer revealed complete obstruction of the distal side of the membranous urethra. (Figure 2D).

This case highlights the importance of recognizing the risk of pelvic fractures in elderly patients who fall while riding a bicycle, even if they can walk independently. Physicians are unaware that pelvic fractures in the elderly due to low‐energy trauma or even minor but repetitive loading on osteoporotic bone^1^ can result in urethral injuries followed by intramuscular abscesses in the hip region. Falls are becoming more common in the elderly, and emergency room evaluations of fall‐related injuries are increasing.^2^ Physicians should pay close attention to enable early detection of acute pelvic fractures using CT and magnetic resonance imaging in elderly patients who have fallen from their bicycles.^3^

## AUTHOR CONTRIBUTIONS

**Yoko Matsumoto:** Conceptualization; data curation; investigation; project administration; writing – original draft. **Keisuke Tanno:** Data curation; investigation; visualization; writing – review and editing. **Yuhei Nakamura:** Data curation; investigation; project administration; writing – review and editing. **Kohei Hamamoto:** Investigation; writing – review and editing. **Hanako Yoshihara:** Investigation; supervision. **Takahiko Fukuchi:** Investigation; supervision. **Noriko Oyama‐Manabe:** Supervision; writing – review and editing. **Hitoshi Sugawara:** Conceptualization; data curation; investigation; project administration; supervision; visualization; writing – original draft; writing – review and editing.

## FUNDING INFORMATION

No funding was obtained for this study.

## CONFLICT OF INTEREST STATEMENT

The authors declare no competing interests.

## ETHICS STATEMENT

Ethical approval from the institutional review board of the authors' institution was not required for this case report.

## PATIENT CONSENT

Written informed consent was obtained from the patient.

## Data Availability

The datasets generated and analyzed are available upon reasonable request to the corresponding author.
